# Effect of long-term antihypertensive treatment on cerebrovascular structure and function in hypertensive rats

**DOI:** 10.1038/s41598-023-30515-0

**Published:** 2023-03-01

**Authors:** Daphne M. P. Naessens, Judith de Vos, Edo Richard, Micha M. M. Wilhelmus, Cornelis A. M. Jongenelen, Edwin R. Scholl, Nicole N. van der Wel, Johannes A. Heijst, Charlotte E. Teunissen, Gustav J. Strijkers, Bram F. Coolen, Ed VanBavel, Erik N. T. P. Bakker

**Affiliations:** 1 Amsterdam UMC Location University of Amsterdam, Biomedical Engineering and Physics, Meibergdreef 9, 1105 AZ Amsterdam, The Netherlands; 2 Amsterdam Cardiovascular Sciences, Microcirculation, Amsterdam, The Netherlands; 3 Amsterdam Neuroscience, Neurovascular Disorders, Amsterdam, The Netherlands; 4 Amsterdam UMC Location University of Amsterdam, Public and Occupational Health, Amsterdam, The Netherlands; 5 Department of Neurology, Donders Institute for Brain, Cognition and Behaviour, Radboud University Medical Center, Nijmegen, The Netherlands; 6 Amsterdam UMC Location Vrije Universiteit Amsterdam, Anatomy and Neurosciences, Amsterdam, The Netherlands; 7 Amsterdam Neuroscience, Neurodegeneration, Amsterdam, The Netherlands; 8 Amsterdam UMC Location University of Amsterdam, Medical Biology, Electron Microscopy Center Amsterdam, Amsterdam, The Netherlands; 9 Amsterdam UMC Location Vrije Universiteit Amsterdam, Neurochemistry Laboratory, Clinical Chemistry, Amsterdam, The Netherlands; 10 Amsterdam Neuroscience, Neuroinfection and -Inflammation, Amsterdam, The Netherlands

**Keywords:** Neuro-vascular interactions, Hypertension

## Abstract

Midlife hypertension is an important risk factor for cognitive impairment and dementia, including Alzheimer’s disease. We investigated the effects of long-term treatment with two classes of antihypertensive drugs to determine whether diverging mechanisms of blood pressure lowering impact the brain differently. Spontaneously hypertensive rats (SHR) were either left untreated or treated with a calcium channel blocker (amlodipine) or beta blocker (atenolol) until one year of age. The normotensive Wistar Kyoto rat (WKY) was used as a reference group. Both drugs lowered blood pressure equally, while only atenolol decreased heart rate. Cerebrovascular resistance was increased in SHR, which was prevented by amlodipine but not atenolol. SHR showed a larger carotid artery diameter with impaired pulsatility, which was prevented by atenolol. Cerebral arteries demonstrated inward remodelling, stiffening and endothelial dysfunction in SHR. Both treatments similarly improved these parameters. MRI revealed that SHR have smaller brains with enlarged ventricles. In addition, neurofilament light levels were increased in cerebrospinal fluid of SHR. However, neither treatment affected these parameters. In conclusion, amlodipine and atenolol both lower blood pressure, but elicit a different hemodynamic profile. Both medications improve cerebral artery structure and function, but neither drug prevented indices of brain damage in this model of hypertension.

## Introduction

Hypertension is an important risk factor for both vascular dementia and Alzheimer’s disease. Particularly midlife hypertension is associated with an increased risk of cognitive decline and dementia later in life^1^. Even though this association between hypertension and neuropathological features has been described in multiple studies^2^, the causal relationship between elevated blood pressure and neurodegeneration is complex and still not fully understood.

Despite the lack of mechanistic insight, hypertension has been recognized as an important treatable condition that could potentially prevent or delay the onset of dementia-related cognitive deficits^2^. To date, several observational and randomized controlled studies have described a protective effect of antihypertensive medication on cognitive decline^3^. This revealed that certain classes appear to decrease the risk for dementia more effectively than others, suggesting that the protective effects may not result from reducing the blood pressure only, but may include mechanisms beyond blood pressure lowering^4,5^. However, direct comparison of different antihypertensive medication classes is scarce, and follow-up times in these studies may have been too short to detect clinical effects^2,4^.

In this study, we tested the hypothesis that the benefit of antihypertensive medication on the brain is class specific. Currently, antihypertensive treatment includes, among others, the prescription of calcium channel blockers or beta blockers, of which amlodipine and atenolol, respectively, are representative for these classes^6^. Previous work in patients revealed that these drugs yield contrasting results with respect to correction of vascular structure and restoration of endothelial function in peripheral arteries^7^, but their impact on the brain vasculature is less clear. Specifically, we anticipated that the vasodilator properties of amlodipine would provide additional benefit regarding cerebral blood flow, cerebrovascular structure and function, over atenolol, which may lower blood pressure through a decrease in cardiac output. To address this hypothesis, we used the spontaneously hypertensive rat (SHR) as a model for hypertension.

Most previous studies used young adult rats, with only a relatively short period of antihypertensive treatment^8–10^. These studies may therefore not fully capture the impact of chronic hypertension and long-term use of antihypertensive drugs. Therefore, in this study we followed up on treatment for one year. We measured systemic parameters such as blood pressure and heart rate, hemodynamic data at the level of carotid artery as major input of blood flow to the brain, and structure and function of the cerebral arteries. Finally, we addressed the implications of treatment for the brain by measuring tissue and cerebroventricular volume, and several other parameters of neurodegeneration.

## Results

### Amlodipine and atenolol similarly lower blood pressure in spontaneously hypertensive rats

**Figure 1:**
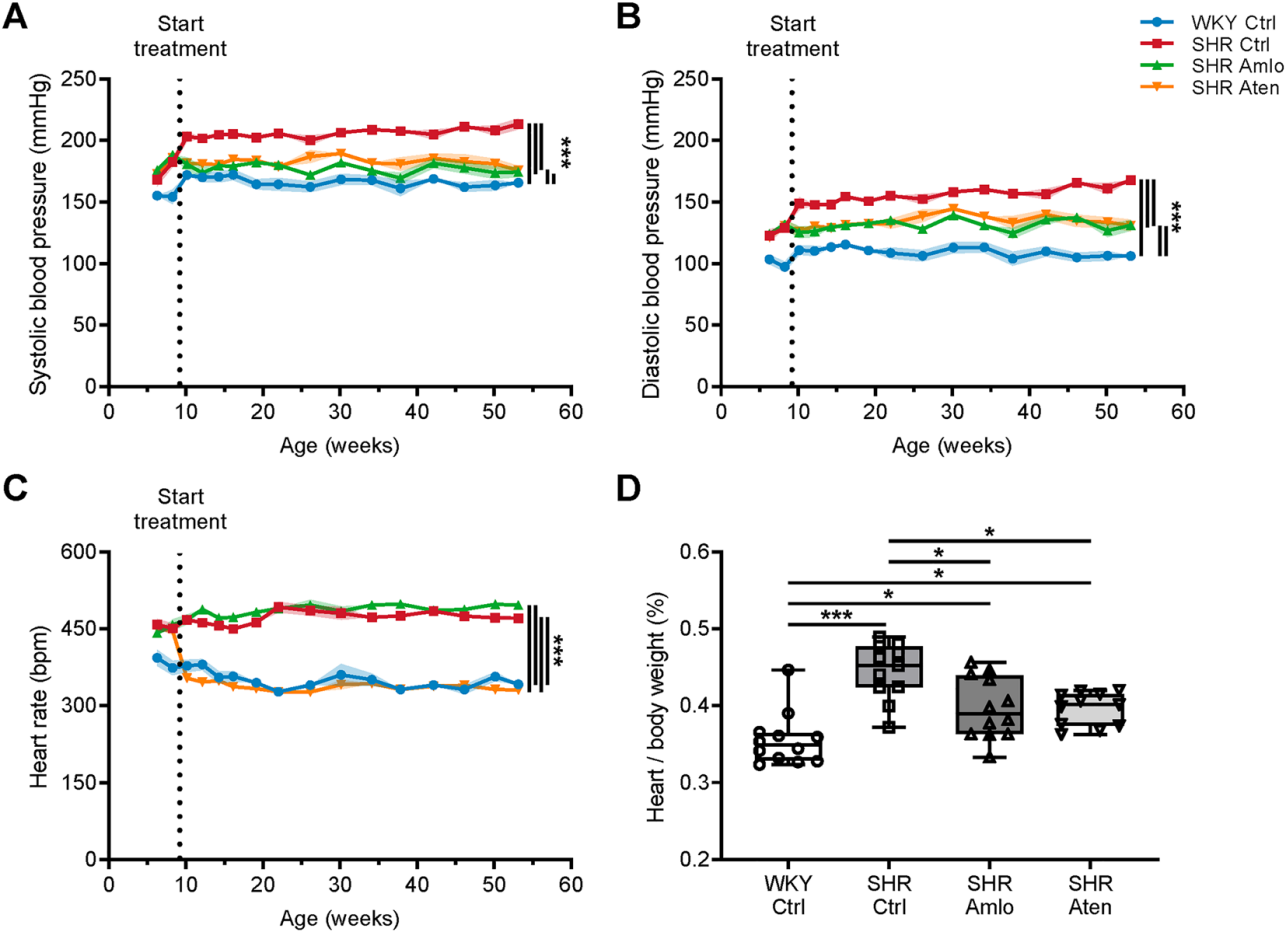
Blood pressure and heart rate before and during treatment with antihypertensive drugs, and heart-to-body weight ratio. (**A**,**B**) During treatment, both systolic and diastolic blood pressure were significantly lower in SHR treated with amlodipine and atenolol when compared to the untreated hypertensive control group. (**C**) Heart rate was significantly lower in atenolol treated SHR and was comparable to the heart rate of normotensive control rats. (**D**) Heart weights were normalized to the body weight. Untreated SHR showed higher heart-to-body weights as compared to all other groups, which was partially normalized by treatment with amlodipine and atenolol. n = 12 for WKY Ctrl and SHR Amlo, and n = 11 for SHR Ctrl and SHR Aten. ***p ≤ 0.001, *p ≤ 0.05 (**A**–**C** mixed-effects model with Bonferroni correction, **D** one-way ANOVA with Bonferroni correction).

Both amlodipine and atenolol significantly lowered systolic and diastolic blood pressures to a similar extent, but blood pressures remained higher than in normotensive rats (Fig. 1A,B). As anticipated, only treatment with atenolol lowered heart rate to a level comparable to WKY (Fig. 1C). At the end of the treatment period, hearts were dissected and weighed to assess the effects of antihypertensive treatment on this organ. As shown in Fig. 1D, untreated SHR had a significantly higher heart-to-body ratio when compared to all other groups, indicating cardiac hypertrophy in hypertension. Even though ratios were significantly higher in amlodipine and atenolol treated animals as compared to normotensive WKY, antihypertensive treatment reduced hypertrophy of the heart. Alongside these measurements, body weight and water intake were monitored (Supplementary Fig. 1). This revealed small differences in growth rate between groups. Water consumption was higher in WKY at a younger age, but upon maturation gradually declined to a level similar to SHR.

### Hemodynamic parameters are differentially affected by antihypertensive treatment

**Figure 2:**
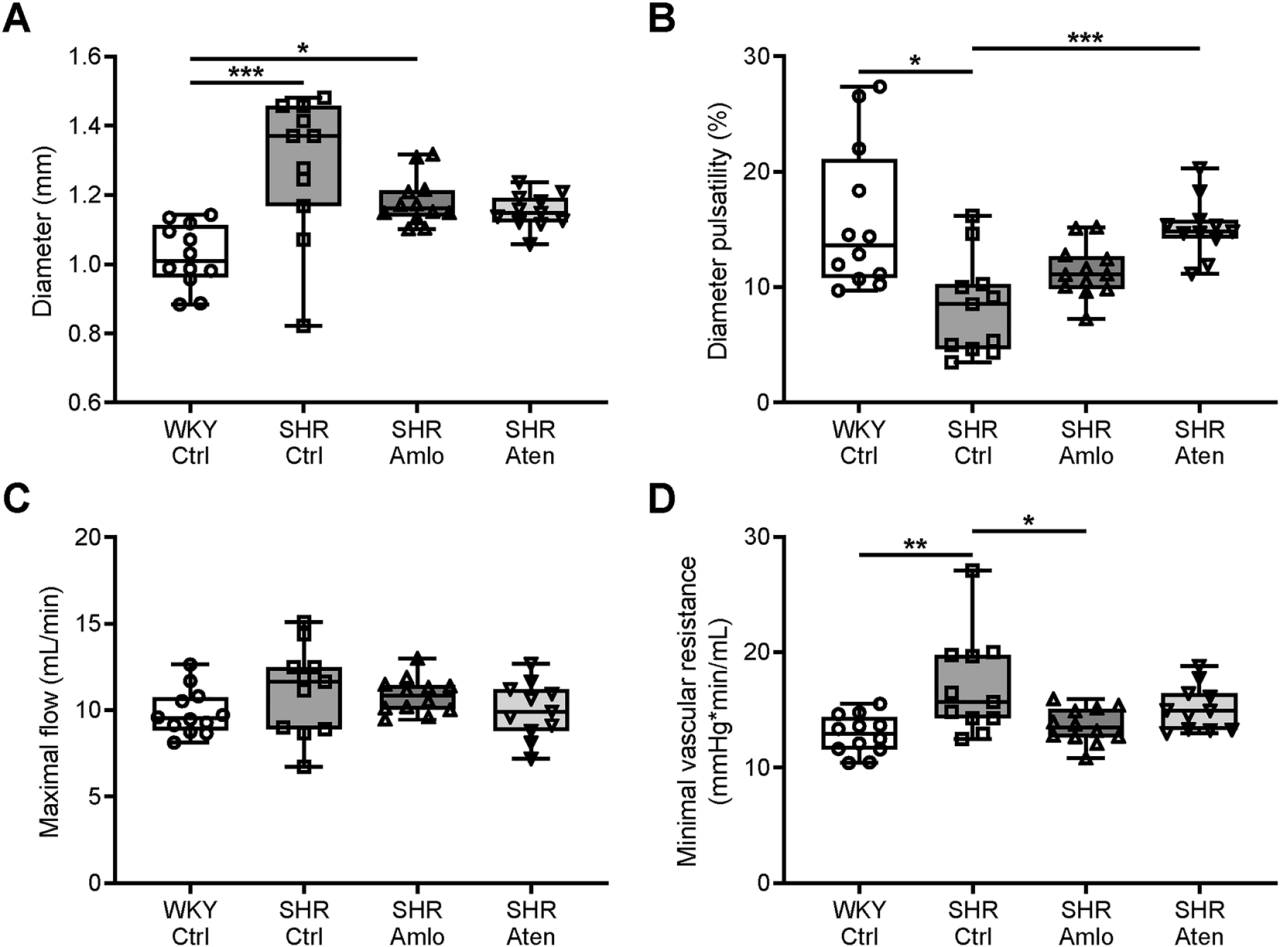
Hemodynamic and vascular parameters as measured using ultrasound imaging. (**A**) Common carotid artery diameter was significantly larger in untreated and amlodipine treated SHR when compared to normotensive WKY rats. (**B**) Control SHR showed a reduced diameter pulsatility, while treatment with atenolol increased diameter pulsatility to a similar level as in WKY. (**C**) Maximal flow, as measured during hypercapnia, was not different among the four groups. (**D**) Minimal vascular resistance was significantly increased in control SHR under hypercapnic conditions. This was partially corrected in amlodipine, but not in atenolol treated SHR. n = 12 for WKY Ctrl and SHR Amlo, and n = 11 for SHR Ctrl and SHR Aten. ***p ≤ 0.001, **p ≤ 0.01, *p ≤ 0.05 (**A**,**C**,**D** one-way ANOVA with Bonferroni correction, and **B** Kruskal–Wallis test with Dunn’s correction).

At the end of the antihypertensive treatment period, ultrasound imaging of the common carotid artery was performed to determine specific vascular and hemodynamic parameters. The mean diameter was significantly smaller in normotensive WKY when compared to untreated SHR. Treatment with amlodipine and atenolol both appeared to partially reduce the mean diameter, although these differences did not reach statistical significance (Fig. 2A). Diameter pulsatility calculations were based on systolic and diastolic diameters, and were reduced in untreated and amlodipine treated SHR. Treatment with atenolol significantly enhanced diameter pulsatility, which was comparable to that in WKY (Fig. 2B). After these measurements, hypercapnia was induced to evoke maximal vasodilation. As shown in Fig. 2C, the resulting maximal blood flow was similar among all groups. From these blood flow and the blood pressure data, the minimal vascular resistance was calculated. Untreated SHR showed an increased minimal vascular resistance when compared to normotensive WKY. This was partially normalized by treatment with amlodipine, but not atenolol (Fig. 2D).

### Cerebrovascular structure and function are partially improved by antihypertensive therapy

**Figure 3:**
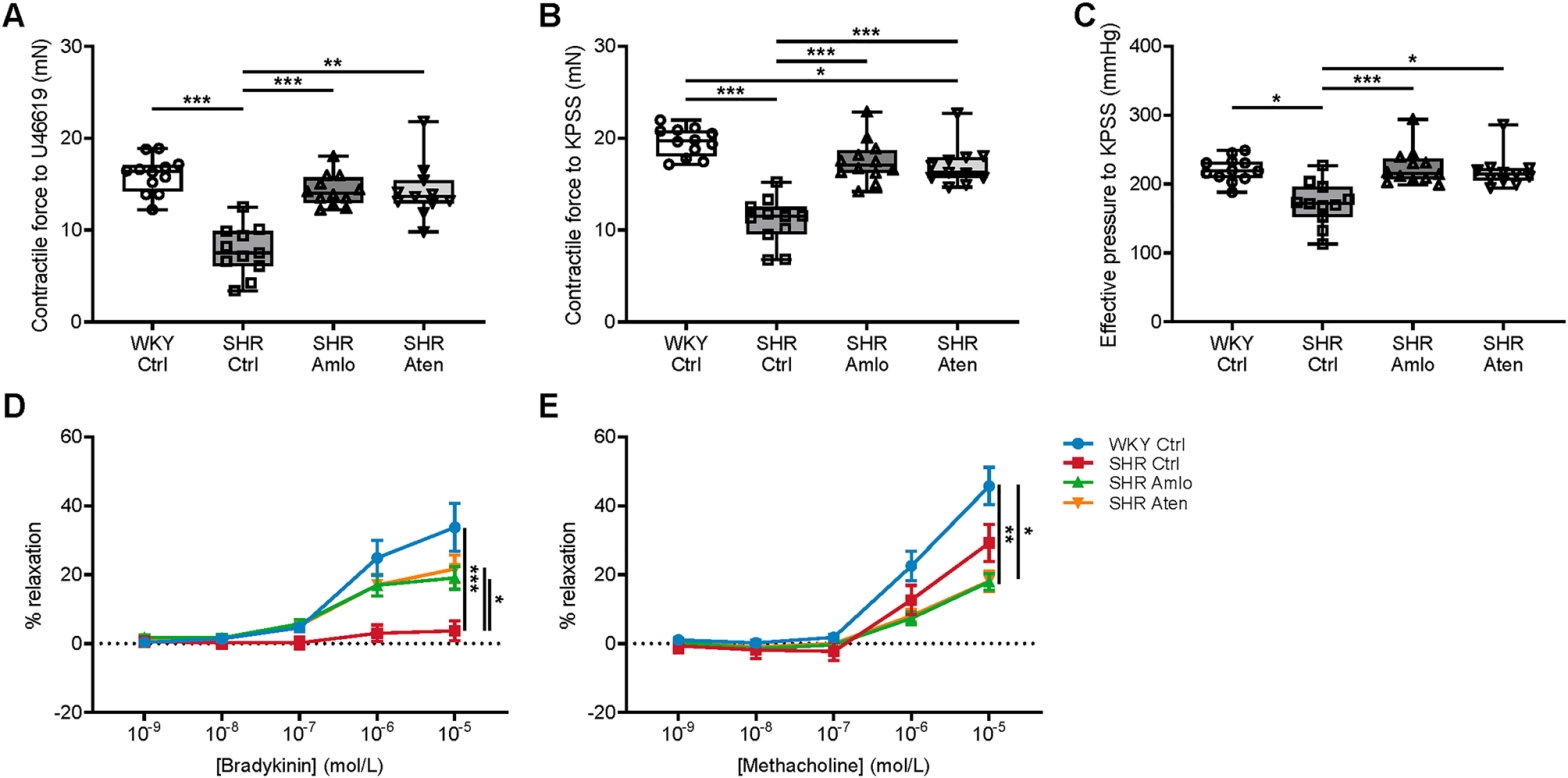
Smooth muscle cell and endothelial function of the basilar artery. (**A**,**B**) To determine smooth muscle cell function, basilar artery segments were constricted with U46619 and KPSS. The contractile force generated in response to these compounds was lowest in untreated SHR. Both treatment with amlodipine and atenolol significantly increased the contractile force, and nearly restored this to the level of WKY. (**C**) The effective pressure equivalent to the contraction with KPSS was significantly lower in untreated SHR when compared to WKY and treated SHR. (**D**,**E**) Endothelial function was determined by assessing the concentration-dependent relaxation to bradykinin and methacholine. (**D**) Control SHR showed an impaired relaxation to bradykinin, while WKY, amlodipine and atenolol treated SHR demonstrate a comparable relaxation. (**E**) In response to methacholine, treated animals showed a reduced relaxation when compared to WKY and untreated SHR. n = 12 for WKY Ctrl and SHR Amlo, and n = 11 for SHR Ctrl and SHR Aten. ***p ≤ 0.001, **p ≤ 0.01, *p ≤ 0.05 (**A**–**C** one-way RM ANOVA with Bonferroni correction, **D**–**E** two-way RM ANOVA with Bonferroni correction).

Besides the assessment of hemodynamic parameters in vivo, vascular structure and function were investigated ex vivo in isolated cerebral arteries using different techniques. A wire myograph setup was used to study smooth muscle cell and endothelial function in the basilar artery. The contractile force of the vessel segments was determined after stimulation with the thromboxane analogue U46619 (Fig. 3A), as well as after stimulation with KPSS (Fig. 3B). This revealed that in both cases, untreated SHR showed lower contractile forces than normotensive WKY and treated SHR. Treatment with amlodipine and atenolol significantly increased the contractile force, and restored it to nearly normotensive levels. Insufficient contractile force could lead to contractile failure in periods of high blood pressure. To test whether the vessels are sufficiently able to contract against high pressure, we determined the equivalent effective pressure during contraction with KPSS (Fig. 3C), based on the Laplace relation. The calculated effective pressure was lowest in control SHR and was nearly normalized to the level of WKY in animals treated with either amlodipine or atenolol. After pre-contraction with U46619, endothelial function was assessed from the concentration-dependent relaxation to bradykinin and methacholine. Figure 3D shows strongly impaired relaxation to bradykinin in untreated SHR, whereas WKY and treated SHR demonstrated a comparable dose-dependent relaxation to this compound. Remarkably, the response to methacholine was impaired in amlodipine and atenolol treated SHR as compared to the normotensive control group, whereas the response in the untreated hypertensive control group was not statistically different from any other group (Fig. 3E).

**Figure 4:**
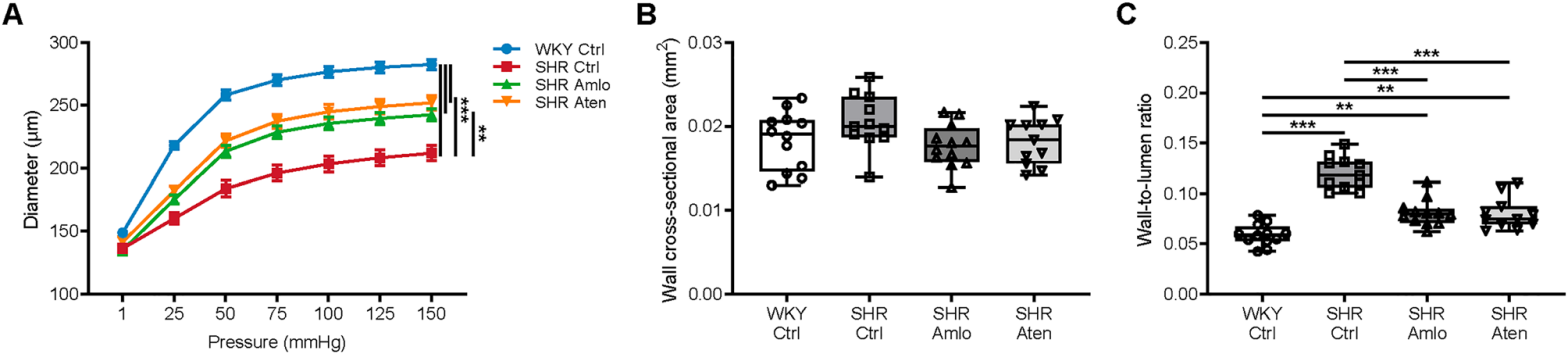
Remodelling of the superior cerebellar artery. (**A**) Diameter of the superior cerebellar artery was determined by measurement of the intraluminal diameter over a pressure range of 1–150 mmHg. Arteries from untreated SHR showed a smaller diameter when compared to control WKY. Amlodipine and atenolol treatment increased the diameter to the same extent, but did not normalize to normotensive levels. (**B**) No differences were observed in the wall cross-sectional area between the four groups. (**C**) Untreated, control SHR showed an increased wall-to-lumen ratio, which was partially normalized by treatment with amlodipine and atenolol. n = 12 for WKY Ctrl and SHR Amlo, and n = 11 for SHR Ctrl and SHR Aten. ***p ≤ 0.001, **p ≤ 0.01 (**A** two-way RM ANOVA with Bonferroni correction, **B**,**C** one-way ANOVA with Bonferroni correction).

Cerebrovascular function and structure were further studied under fully dilated conditions in superior cerebellar arteries in a pressure myograph. The diameter of the arteries was determined by measuring the intraluminal diameter over a pressure range of 1 to 150 mmHg. As shown in Fig. 4A, vessels from untreated, hypertensive animals showed a significant reduction in diameter, particularly at higher pressure levels. This was the result of a reduction in distensibility of the vessels at lower pressure levels (Supplementary Fig. 2). Treatment with amlodipine and atenolol increased the diameter to the same extent, but did not normalize it to normotensive values. Figure 4B shows that there were no differences between the four groups of the wall cross-sectional area (CSA), a measure of hypertrophy. However, the wall-to-lumen ratio was significantly increased in untreated, control SHR as compared to normotensive WKY and treated SHR. Amlodipine and atenolol treated SHR had similar wall-to-lumen ratios, both significantly lower than the hypertensive control group (Fig. 4C). Together, these data show a decreased luminal diameter with preserved wall CSA, suggesting that the remodelling of the superior cerebellar artery in hypertension is inward and of a eutrophic nature.

**Figure 5:**
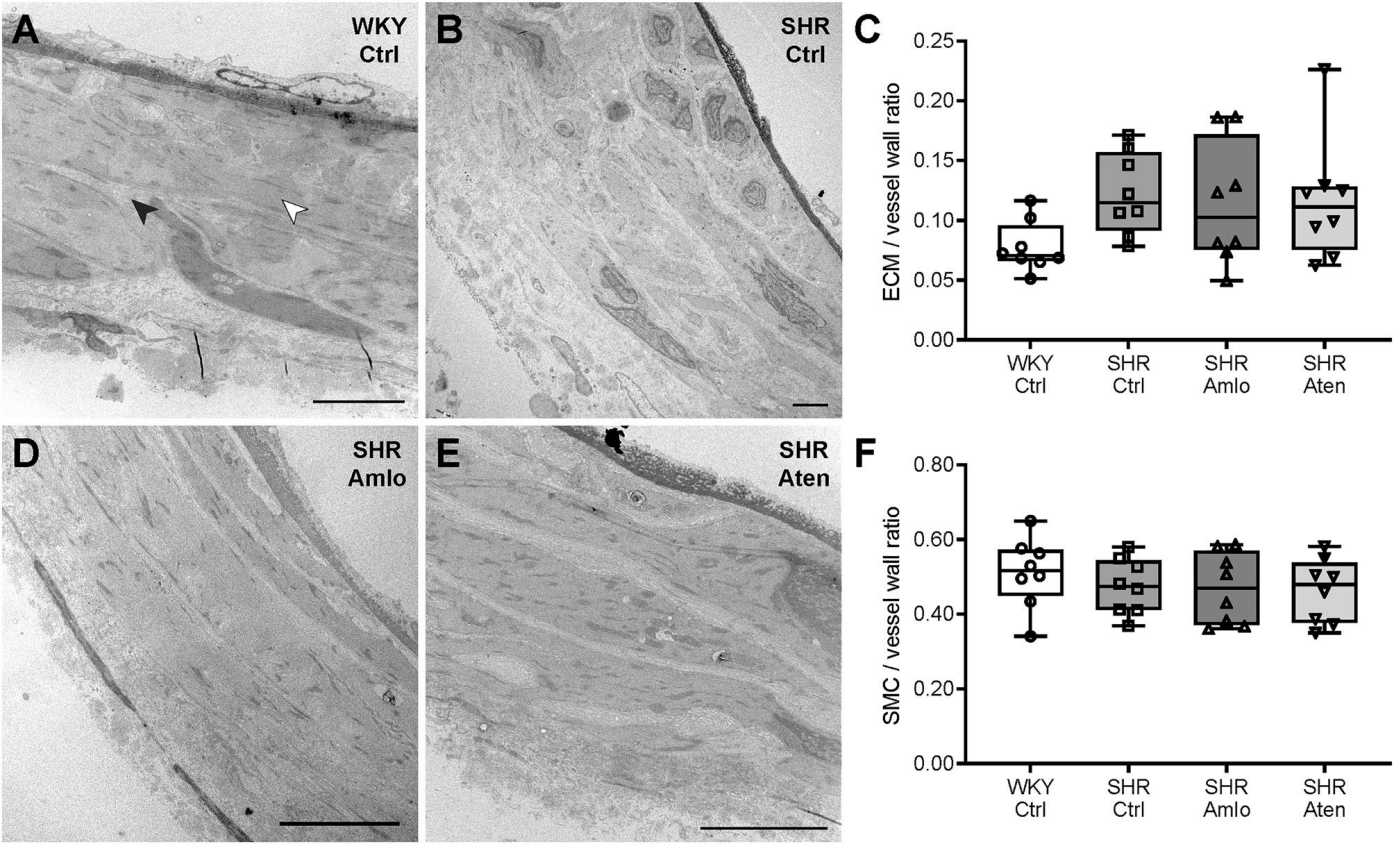
Extracellular matrix and smooth muscle cell quantification in superior cerebellar arteries. (**A**,**B**,**D**,**E**) Representative cross-sectional images of the superior cerebellar artery wall of normotensive WKY, untreated SHR, amlodipine and atenolol treated SHR, respectively, in which the lumen of the artery is shown at the top. The black arrow indicates the extracellular matrix (ECM), whereas the white arrow indicates a smooth muscle cell (SMC). (**C**) The ECM/vessel wall ratio was not different between the four groups. (**F**) SMC/vessel wall ratios did also not differ between any of the groups. n = 8 for WKY Ctrl, SHR Ctrl, SHR Amlo, and SHR Aten. (**C**,**F** one-way RM ANOVA with Bonferroni correction). Scale bar represents 5 µm.

Since measurements on the mechanical properties of the superior cerebellar artery revealed differences between normotensive WKY, untreated, and treated SHR, transmission electron microscopy was performed on a subset of pressurized vessel segments to evaluate arterial ultrastructure. The extracellular matrix and smooth muscle cell quantity were assessed in three different locations of the artery, and were determined as fraction of the total vessel wall. In Fig. 5, representative cross-sectional images of the superior cerebellar artery from a normotensive WKY (Fig. 5A), untreated SHR (Fig. 5B), amlodipine treated SHR (Fig. 5D), and atenolol treated SHR (Fig. 5E) are shown. The extracellular matrix/vessel wall ratio tended to be higher in control SHR when compared to normotensive WKY. This was due to focal areas of extracellular matrix deposition. However, no statistical differences were observed between any of the groups (Fig. 5C). In addition, no differences in the smooth muscle cell/vessel wall ratio were found between the four groups, although vessels from untreated SHR often showed a less organized morphology (Fig. 5F).

### Antihypertensive treatment does not affect brain volume

**Figure 6:**
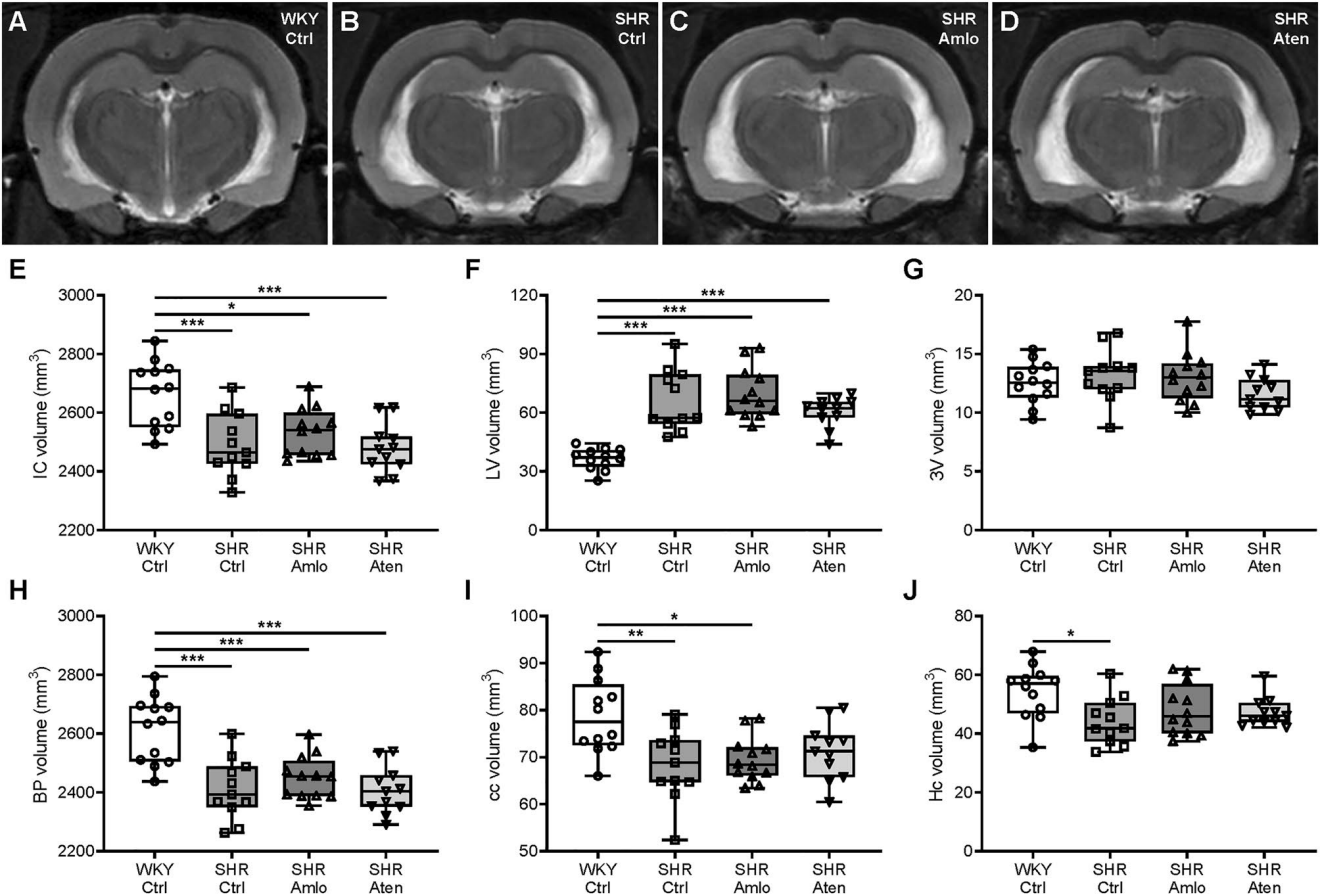
Volume measurements of the brain and specific anatomical structures. (**A**–**D**) Representative single slices of the brains of normotensive WKY, untreated SHR, amlodipine and atenolol treated SHR, respectively. (**E**) Total intracranial volumes were significantly smaller in all SHR groups when compared to control WKY. (**F**) Lateral ventricles were substantially enlarged in both untreated and treated rats. (**G**) No differences were noted in the third ventricle volume between the four groups. (**H**) The brain parenchyma volume was significantly decreased in untreated, control SHR, as well as amlodipine and atenolol treated SHR. (**I**,**J**) The corpus callosum volume was smaller in control and amlodipine treated SHR, while the hippocampal volume was significantly decreased in untreated hypertensive rats only. n = 12 for WKY Ctrl and SHR Amlo, and n = 11 for SHR Ctrl and SHR Aten. ***p ≤ 0.001, **p ≤ 0.01, *p ≤ 0.05 (**E**–**J** one-way ANOVA with Bonferroni correction). *IC* intracranial, *LV* lateral ventricle, *3V* third ventricle, *BP* brain parenchyma, *cc* corpus callosum, *Hc* hippocampus.

T2w anatomical scans were made to assess the volume of the whole brain and specific anatomical structures, including lateral ventricles, corpus callosum and hippocampus. Figure 6A–D show representative examples of the same stereotaxic coordinates of a single slice in the four groups. Total intracranial brain volumes were significantly smaller in all SHR groups when compared to normotensive animals (Fig. 6E). These data correspond well to the wet brain weights as measured after dissection of the brain following MR imaging (Supplementary Fig. 3). The size of the lateral ventricles was substantially larger in both untreated and treated rats (Fig. 6F), while third ventricle sizes were similar in all groups (Fig. 6G). Based on these measurements, the brain parenchymal volume was calculated by subtracting the total ventricle volume from the intracranial volume. This revealed that SHR had smaller brain tissue volumes, and that antihypertensive treatment did not normalize these volumes (Fig. 6H). The volume of the corpus callosum was significantly smaller in untreated SHR and amlodipine treated SHR when compared to WKY. A similar trend was observed for atenolol treated SHR, although this did not reach statistical significance (Fig. 6I). Lastly, hippocampal volume was smaller in untreated SHR, but not in both treated SHR groups (Fig. 6J).

### Hypertensive rats do not show differences in soluble amyloid-β levels, but do show signs of neuronal damage

**Figure 7:**
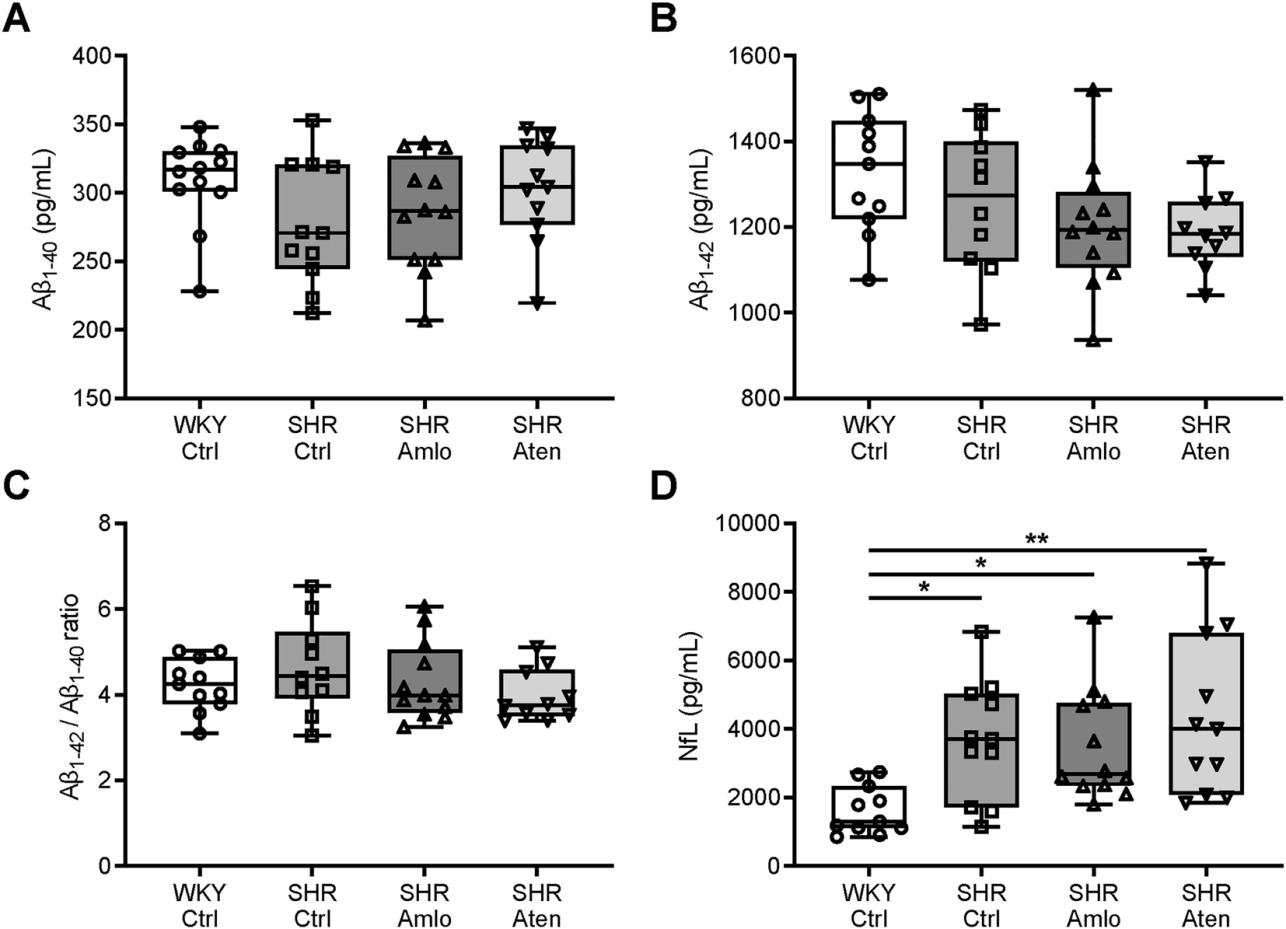
Amyloid-β_1–40_, amyloid-β_1–42_, and neurofilament light expression levels in the brain and CSF. (**A**,**B**) Amyloid-β_1–40_ (Aβ_1–40_) and amyloid-β_1–42_ (Aβ_1–42_) concentrations in brain homogenates were not different between the four groups. (**C**) Additionally, the Aβ_1–40_/Aβ_1–42_ ratios were similar in all groups. (**D**) Neurofilament light (NfL) levels were significantly elevated in the cerebrospinal fluid (CSF) of untreated and treated SHR as compared to WKY. n = 11–12 for WKY Ctrl, n = 10–11 for SHR Ctrl and SHR Aten, n = 12 for SHR Amlo. **p ≤ 0.01, *p ≤ 0.05 (**A**–**C** one-way ANOVA with Bonferroni correction, **D** Kruskal–Wallis test with Dunn’s correction).

**Figure 8:**
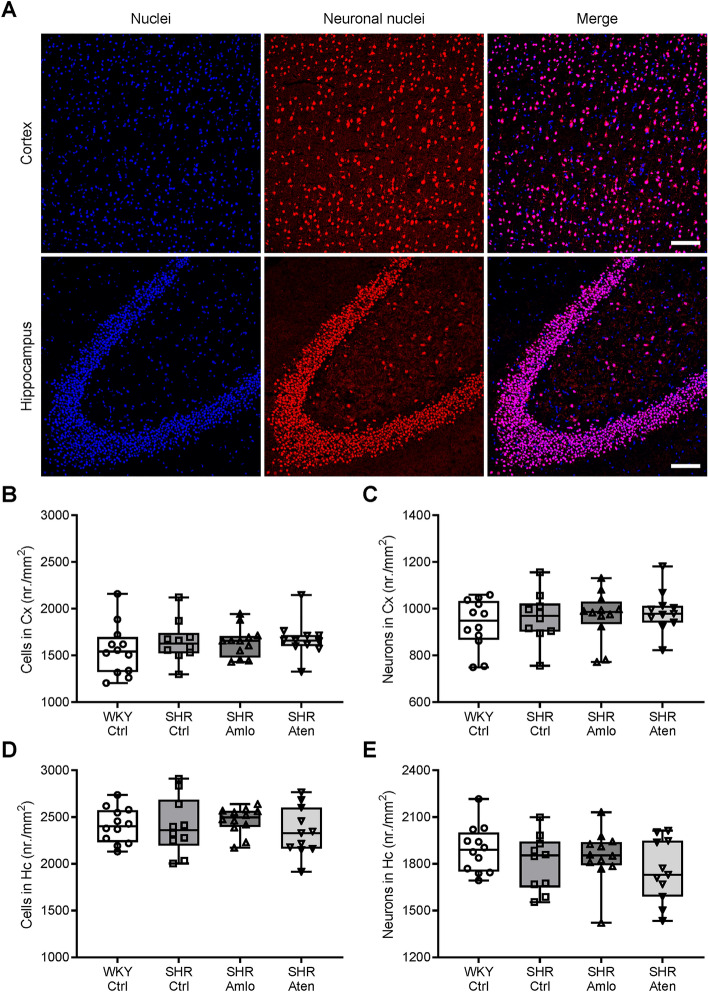
Number of cells and neurons in the cortex and hippocampus. (**A**) Representative images of NeuN (red) immunostaining in the cortex and hippocampus. Cell nuclei are visualised using DAPI staining (blue). (**B**,**C**) The number of cells did not differ between the four groups in the cortex and hippocampus. (**D**,**E**) Additionally, the number of neurons was not different between groups. n = 12 for WKY Ctrl and SHR Amlo, n = 10 for SHR Ctrl, n = 11 for SHR Aten. (**C** Kruskal–Wallis test with Dunn’s correction, **D**–**F** one-way ANOVA with Bonferroni correction). *Cx* cerebral cortex, *Hc* hippocampus. Scale bar represents 100 µm.

To examine effects on soluble amyloid-β (Aβ) levels, axonal damage, and neurodegeneration in treated and untreated SHR, we assessed soluble Aβ and neurofilament light (NfL) levels in brain lysates and CSF samples, respectively. Aβ_1–40_ and Aβ_1–42_ concentrations were measured in the same brain homogenates using ELISA. No significant differences in either Aβ_1–40_ or Aβ_1–42_ levels were found between the four groups (Fig. 7A,B). As shown in Fig. 7C, Aβ_1–42_/Aβ_1–40_ ratios were similar in all groups, indicating that hypertension, with or without treatment with antihypertensive drugs, did not significantly affect soluble Aβ_1–40_ or Aβ_1–42_ levels compared to normotensive rats. In addition, NfL, a biomarker for axonal damage, was measured in the CSF. The levels of this biomarker were significantly elevated in untreated and atenolol treated SHR when compared to WKY. Treatment with amlodipine tended to reduce NfL levels, however this was still significantly different from normotensive values (Fig. 7D). Lastly, neurodegeneration was evaluated by counting the total number of cells and neurons per mm^2^ in the hippocampus and cortex. Brain tissue sections were immunolabelled for NeuN, which specifically visualises neuronal nuclei. As shown in Fig. 8, no differences in the number of cells and neurons were noted between the groups.

## Discussion

In this study, we investigated the long-term effects of hypertension on the brain, a major target of end-organ damage. Furthermore, we studied whether drugs from different classes of antihypertensive medication provide the same level of protection against cerebrovascular dysfunction and brain injury.

Systolic and diastolic blood pressures were lowered to the same extent by amlodipine and atenolol. Heart rate was, as anticipated, reduced in atenolol treated SHR only, reflecting the mode of action of this drug. Since high heart rate is an independent risk factor for cardiovascular disease^11^, the current study allows for untangling of the contribution of blood pressure and heart rate to end-organ damage. A first, straightforward parameter we assessed in this respect was heart weight. We found that hypertension was manifested in an increased heart-to-body-weight ratio, which was similarly reduced in the two treatment groups. Blood pressure and heart weight remained higher than those of the normotensive WKY in both amlodipine and atenolol treated animals. This suggests that cardiac hypertrophy closely correlates with blood pressure, without an apparent interaction with heart rate or other indirect effects of the medication.

The carotid artery represents a major conduit for blood flow from the heart to the brain. Mean arterial diameters were remarkably larger in untreated SHR as compared to normotensive WKY, which was partially normalized in amlodipine and atenolol treated rats. Moreover, we observed a reduced amplitude in diameter oscillations during the cardiac cycle in untreated SHR. These changes confirm previous findings in SHR^12^, and reflect differences in local pressure and mechanical properties between SHR and WKY. Interestingly, the reduction in diameter oscillations was antagonized by treatment with atenolol only. The most likely explanation for this finding is that the reduced heart rate in this group accommodates larger diameter oscillations due to the viscoelastic properties of arteries^13^, but differences in intrinsic mechanical properties cannot be excluded. Notwithstanding the origin, these alterations in hemodynamics may have a number of consequences. Most important of these damping of pressure pulsations may be impaired in untreated SHR. Such pressure pulsations are transmitted to the microcirculation, which is considered detrimental^14,15^.

The minimal resistance of the cerebral vasculature was increased in untreated SHR, which may be attributable to vascular remodelling and/or changes in vascular density. The observation that amlodipine, but not atenolol, antagonized this increase in resistance suggests a favourable effect of this drug with regard to this parameter. This is in agreement with previous data on human peripheral arteries^7^. It is however not clear which part of the vasculature is favourably treated by amlodipine. Our ex vivo analysis of the posterior cerebellar artery showed a similar beneficial effect from amlodipine and atenolol. Possibly, other parts of the vascular bed are differentially affected by the antihypertensive treatment. We focussed on the arteries because morphological and mechanical changes of the resistance arteries are often considered a hallmark of hypertension^16^. Multiple animal studies demonstrated that hypertension is associated with remodelling of the cerebral resistance arteries^17–19^, which was also shown in human studies more recently^20,21^. Generally, hypertension leads to an increased wall-to-lumen ratio, which can be seen as an adaptation to reduce wall stress. However, this response has a negative prognostic value with respect to major cardiovascular events^22^. The increase in wall-to-lumen ratio may result from hypertrophy, inward remodelling, or a combination of these^23^. Inward remodelling without changes in the wall cross-sectional area is known as eutrophic inward remodelling. The question whether certain antihypertensive drugs are more effective than others to correct remodelling has been raised in the past^24^, and for review see Schiffrin^25^, but most of this work investigated peripheral arteries. In the current study, cerebral arteries from untreated SHR showed eutrophic inward remodelling, evidenced by a reduction of the lumen diameter, an increased wall-to-lumen ratio, and an unaltered cross-sectional area of the vessel wall. As indicated above, treatment with amlodipine and atenolol both partially prevented this alteration to a similar extent. The reduction in diameter was evident at higher pressures only, suggesting that a reduction in distensibility at lower pressure levels underlies remodelling in these arteries. Distensibility is defined as the change in diameter with each increase in pressure^26^. Such a reduction in distensibility may result from changes in the extracellular matrix, such as the deposition of collagen. However, ultrastructural analysis of the vessel with electron microscopy did not show significant differences in extracellular matrix content between the groups, suggesting that quantitative differences are small, or other factors such as the rearrangement and cross-linking of these structures may contribute to vascular stiffness^27–29^.

The remodelling of the cerebral arteries was accompanied by impaired contractile function in untreated SHR. As the arteries are free of paracrine tissue after dissection, this reduced contractile function appears intrinsic to the artery. Such reduction in contractile force is surprising, as vessels from SHR need to oppose a higher local pressure. The effective pressures that correspond to the maximal contractile force were lowest in untreated SHR, while treatment with both agents completely reversed this to values observed in WKY. Interestingly, the effective pressures as calculated in isolated cerebral arteries were close to the systemic blood pressures. This finding suggests that arteries of untreated SHR can hardly constrict under the prevailing pressures, which, in turn, may lead to a reduced capability to maintain cerebral autoregulation. It should be acknowledged though, that the calculation of effective pressure was made in the wire myograph setup. Herein the vessels are flattened, which could interfere with the maximal force developed by the muscle cells. In addition to difficulties to achieve constant blood flow to the brain, this may lead to exposure of the microcirculation to an elevated blood pressure. This could, in turn, contribute to endothelial dysfunction as often observed in adult SHR^30^. We therefore assessed endothelial function by measuring the responses to bradykinin and methacholine. This confirmed that cerebral arteries of untreated SHR display marked endothelial dysfunction. Both antihypertensive drugs partially restored endothelial function to bradykinin, but not to methacholine.

Vascular dysfunction may be an intermediate step towards end organ damage. A crude determinant of neurodegeneration is cerebral atrophy, which has been described in both hypertensive patients and animal models of hypertension, often accompanied by cerebroventricular enlargement^31–35^. Ventricular enlargement may be explained by two possible pathological mechanisms. First, it may simply reflect a loss of brain tissue. Second, it may develop due to a hypersecretion of CSF by the choroid plexus. However, previous work from our group showed that hypertensive rats have a normal intracranial pressure and an unaltered CSF production rate^36^. Thus, ventricular dilation more likely results from the substitution of brain tissue loss with CSF. In that respect, antihypertensive therapy could prevent cerebral atrophy and cerebroventricular enlargement. We observed lower brain weights and smaller brain volumes in untreated SHR. Yet, brain weights and intracranial volumes were comparable in untreated and treated SHR, and were paralleled by a similar expansion of the lateral ventricles. This observation indicates that in the current study either blood pressure reduction was insufficient, requires other classes of drugs, or that lower brain weights are not a consequence of hypertension in this model. Previous studies suggest that these parameters are genetically determined in SHR^37,38^, which may be an alternative explanation. The question is whether hypertension is associated with substantial neurodegeneration at all at this age. We found no differences between groups in the levels of amyloid-β_1–40_ and amyloid-β_1–42_. There are few studies describing amyloid-β accumulation in the brain of SHR, suggesting that this is not a prominent feature of this animal model. Using immunohistochemistry, Kurata et al*.*^39^ found increased amyloid-β and phosphorylated tau in SHR in comparison to Wistar rats. A study by Wang et al*.*^40^ in 17 months old rats detected increased amyloid-β in SHR compared to WKY. Schreiber et al*.*^41^ found evidence for amyloid-β and phosphorylated tau deposition in hypertensive rats, but these were stroke-prone SHR, a more aggressive phenotype compared to SHR.

Total cell counts as well neuronal numbers did not reveal any statistical significant difference in both the cortex and hippocampus. However, neurofilament light levels were higher in the CSF of untreated and treated SHR when compared to normotensive WKY. Upon damage of an axon, neurofilament light is released into the extracellular space and CSF. An elevated level of this protein, therefore, is a general indicator for neuronal cell damage and cell death^42^. Thus, although the crude measurements on brain atrophy did not provide definitive proof of neurodegeneration in this model of hypertension, the elevated levels of this biomarker indicate more subtle neuropathological processes. However, neither amlodipine nor atenolol affected this.

In conclusion, treatment of hypertension with either amlodipine or atenolol leads to a clearly distinct outcome regarding the hemodynamic profile of blood flow to the brain. The increase in vascular resistance of the brain vasculature in SHR was prevented by amlodipine only, suggesting a beneficial effect on vascular reserve by this drug. Both antihypertensive drugs partially normalized cerebrovascular structure and function, suggesting that lowering of blood pressure in itself protects from vascular dysfunction. However, neither amlodipine nor atenolol appeared to have beneficial effects on brain atrophy, or axonal damage. Thus, future studies may extend the follow up period of antihypertensive treatment in this rat model, or may include other models of hypertension. In addition, future studies may expand the range of antihypertensive medication classes and incorporate behavioural tasks to assess cognitive function during aging of the animals. The current results provide more insight in the effects of different antihypertensive medication classes on hypertensive brain injury and vascular dysfunction, and may contribute to new perspectives for future research to elucidate the underlying mechanisms of hypertension on cognitive impairment.

## Materials and methods

### Animals

In this study, 12 male normotensive Wistar Kyoto rats (WKY/NCrl) and 36 male spontaneously hypertensive rats (SHR/NCrl) were used. Hypertensive rats were randomly allocated to three different experimental groups: 12 untreated rats (SHR Ctrl), 12 rats treated with amlodipine (SHR Amlo), and 12 rats treated with atenolol (SHR Aten). WKY rats were left untreated and served as a normotensive control group (WKY Ctrl). One untreated SHR and one atenolol-treated SHR died during the study from unknown causes, leaving 11 rats in both of these groups. All animals were obtained from Charles River (Germany) and were 4 weeks of age upon arrival in the animal facility. They were housed in groups of 2 rats per cage on a 12-h light/12-h dark schedule and had ad libitum access to standard laboratory food and drinking water. All experiments were performed in a blinded fashion and were in accordance with the ARRIVE guidelines and European Union guidelines for the welfare of laboratory animals (Directive 2010/63/EU), and were approved by the Academic Medical Center Animal Ethics Committee. All in vivo experiments were carried out under isoflurane inhalation anaesthesia (Pharmachemie B.V.).

### Antihypertensive drug treatment and blood pressure measurements

From 6 weeks of age, rats were weighed once a week and the average water intake was measured per cage. As from this age, blood pressure and heart rate were measured biweekly in conscious rats using a non-invasive tail-cuff system (Kent Scientific). Rats were first accustomed to this procedure by placing them in the restrainer on 4 consecutive days. Ten blood pressure and heart rate measurements were recorded from each rat per measurement session and averaged. From 9 weeks of age, treatment with the antihypertensive agents was initiated. Amlodipine besylate (5 mg/kg/day, Pfizer) and atenolol (50 mg/kg/day, Sigma-Aldrich) were dissolved in the drinking water. As a stable effect of the antihypertensive agents was observed after 7 weeks of treatment, blood pressure and heart rate were measured monthly from this time point on. Animals were treated until 55.1 ± 0.2 weeks of age.

### Ultrasound imaging

A Vevo 2100 Imaging System (FUIJIFILM VisualSonics, Amsterdam, The Netherlands) was used to visualise and measure hemodynamic parameters in the left common carotid artery. Animals were anaesthetized with 1.8–2.0% isoflurane (Pharmachemie B.V.) in a mixture of 0.4 L/min medical air and 0.4 L/min O_2_. After induction of general anaesthesia, rats were placed on the physiological monitoring unit via which body temperature was measured through a rectal probe and maintained at 36–37 °C. Ophthalmic ointment (Duratears^®^, Alcon) was applied to prevent dryness of the eyes. A butterfly catheter (22G, BD Venflon™) was inserted in the tail vein to collect blood samples during the ultrasound imaging procedure. Respiration rate and amplitude were monitored and recorded using a Moku:Lab device (Liquid Instruments) that was connected to a pressure balloon placed on the animal’s abdomen. The hair on the left side of the neck was removed with depilatory cream (Nair) and warm ultrasound gel was applied to the skin for imaging. An MS-550D transducer was used for ultrasound imaging at a frequency of 40 MHz. B-Mode imaging was used to locate the left common carotid artery in the longitudinal view. Then, M-Mode imaging was used to measure movement of the vessel wall over time. The Color Doppler Mode and Pulsed Wave (PW) Doppler Mode were subsequently used to visualise and determine blood flow velocity in the centre of the carotid artery. The ultrasound beam was placed perpendicular to the carotid artery in an angle of 55° or smaller to accurately measure the PW Doppler signal. After acquiring M-Mode and PW Doppler Mode images under normal isoflurane anaesthesia, these measurements were repeated during hypercapnia. While keeping the transducer in place, carrier gases for the isoflurane anaesthesia were replaced for carbogen gas (5% CO_2_/95% O_2_) at a flow rate of 1 L/min. An increase in the respiration rate and pCO_2_ as measured using the RAPIDPoint^®^ 500 system (Siemens) indicated the induction of the hypercapnic response (data not shown). The PW Doppler signal was first recorded to determine blood flow velocity at the same position as during normocapnia and was followed by B-Mode and M-Mode imaging. After acquisition of these images under hypercapnic conditions, the carrier gas was switched back to 0.4 L/min medical air and 0.4 L/min O_2_ with maintained isoflurane anaesthesia. Vevo LAB software was used to assess the systolic, diastolic, and mean diameters, as well as the mean blood flow velocity. These data were used to calculate diameter pulsatility under baseline conditions, and maximal blood flow and minimal vascular resistance during hypercapnia by the use of the following equations:

$${\mathrm{Pulsatility }} ({\%})= \frac{{\mathrm{d}}_{\mathrm{Systole}} - {\mathrm{d}}_{\mathrm{Diastole}}}{{\mathrm{d}}_{\mathrm{Diastole}}} \times 100$$

where d_Systole_ is the diameter during systole and d_Diastole_ is the diameter during diastole.

$${\mathrm{Q}}_{\mathrm{Maximal}} ({\mathrm{mL}}{\cdot {\mathrm{min}}}^{-1})=(\uppi \times {\mathrm{r}}^{2}) \times\left (\frac{\mathrm{v}}{2} \right)$$

where Q_Maximal_ is the maximal blood flow, r is the radius, and v is the time-averaged velocity in the centre of the carotid artery during hypercapnia.

$${\mathrm{SVR }}({\mathrm{mmHg}}\cdot {\mathrm{min}}\cdot {\mathrm{mL}}^{-1})=\frac{{\mathrm{MAP}}-{\mathrm{CVP}}}{{\mathrm{Q}}_{\mathrm{Maximal}}}$$

where SVR is the minimal systemic vascular resistance, MAP is the mean arterial pressure averaged over the last three blood pressure measurements, CVP is the central venous pressure, and Q_Maximal_ the maximal blood flow as previously calculated.

### MR imaging and analysis

After ultrasound imaging, rats were kept anaesthetized using isoflurane and rapidly transferred to the MR system. MR imaging was performed on a 7 T small animal MRI system (MR Solutions, Guildford, United Kingdom) using a rat head coil (MR Solutions). Animals were positioned prone on the MRI cradle and the head was immobilized with a bite bar and ear bars. Maintenance anaesthesia was applied via the nose mask and was kept between 1.8–3.0% in a mixture of 0.4 L/min medical air and 0.4 L/min O_2_. Respiration rate was monitored using a pressure balloon placed at the rat’s abdomen and was maintained a rate of 45–55 breaths per minute by adjusting the isoflurane concentration. Body temperature was maintained at 36–37 °C via the heated animal bed and was measured with a rectal probe.

Scout scans were made to localise the brain and to plan the position of the slices for the subsequent scans. To determine the volume of the whole brain as well as different anatomical structures, coronal multi-slice T2-weighted (T2w) turbo spin-echo images were acquired with the following parameters: TR/TE = 4000/45 ms, flip angle = 90°, echo-train length ETL = 7, FOV = 35 × 35 mm^2^, matrix size = 256 × 256, slice thickness = 1 mm, number of slices = 26, NSA = 4, total acquisition time = 9 min.

Images were analysed as described in the study by Naessens et al*.*^35^. In short, to quantify the whole brain volume as well as the volume of a number of specific anatomical structures, multi-slice coronal T2w images were manually delineated by an experienced observer (DMPN) using ITK-SNAP (version 3.8.0).

### Tissue and fluid collection

At the end of the MR imaging protocol, rats were kept anaesthetised in order to collect a CSF sample. A small skin incision was made at the animal’s back of the neck and the muscles were separated to reach the cisterna magna. A 30-gauge needle (BD Microlance™) connected to a U-100 insulin syringe (BD Micro-Fine™) with polythene tubing (Smiths Medical) was inserted in the cisterna magna, and a CSF sample was collected by gentle aspiration. Rats were subsequently killed by exsanguination under deep isoflurane anaesthesia.

The heart and brain were carefully dissected and weighed. The brain was then transferred to cold MOPS-buffered physiological saline solution (PSS, 145 mM NaCl, 4.7 mM KCl, 1.17 mM MgSO_4_, 1.2 mM NaH_2_PO_4_, 2.0 mM CaCl_2_, 5.0 mM glucose, 2.5 mM pyruvate, pH 7.35). Both left and right superior cerebellar arteries and the basilar artery were dissected, and used for analysis in a pressure myograph or wire myograph setup, respectively. The brain was subsequently divided into different coronal slices using an adult rat brain slicer (Zivic Instruments). One slice of the middle part containing the hippocampus was embedded in TissueTek^®^, whereas another slice of this same part was snap frozen in liquid nitrogen. All samples were stored at − 80 °C until further use.

### Wire myography

To assess endothelial function in cerebral arteries, two segments of the basilar artery were mounted in a wire myograph setup (610 M, Danish Myo Technology, Aarhus, Denmark). Vessels were allowed to equilibrate to 37 °C and were normalized in Ca^2+^-free MOPS-buffered PSS to obtain the optimum distension for force generation^43^. This solution was replaced for PSS (119 mM NaCl, 4.7 mM KCl, 1.18 mM KH_2_PO_4_, 1.17 mM MgSO_4_, 0.026 mM EDTA, 5 mM HEPES, 25 mM NaHCO_3_, 1.6 mM CaCl_2_, 5.6 mM glucose, pH 7.35) and gassed with a mixture of 95% air and 5% CO_2_. Vessels were then exposed twice to 125 mmol/L potassium solution (KPSS) to test for viability and measure the maximum force to this solution. After rinsing with PSS, basilar artery segments were preconstricted with 3 · 10^–6^ µmol/L thromboxane A_2_ agonist U46619 (Sigma-Aldrich). The non-selective muscarinic receptor agonist methacholine was added in a cumulative manner with concentrations ranging from 1 · 10^–9^ to 1 · 10^–5^ mol/L to test endothelium-dependent relaxation to this compound. Methacholine was washed out of the chamber with PSS and vessels were allowed to recover for at least 15 min. Basilar artery segments were again preconstricted with U46619 and a second concentration–response curve was obtained to assess endothelial function using bradykinin. This compound was added cumulatively to the chamber in the concentration range from 1 · 10^–9^ to 1 · 10^–5^ mol/L. The average of the two basilar artery segments was used for the concentration–response curves to both methacholine and bradykinin. These measurements were also used for the assessment of the effective pressure upon constriction using KPSS, based on the following equations^43^:

$${\mathrm{T}}= \frac{{\mathrm{F}}_{\mathrm{KPSS}}}{2\times {\text{vessel length}}}$$

where T is the wall tension and F_KPSS_ the active force upon constriction with KPSS.

$${\mathrm{P}}= \frac{\mathrm{T}}{{\mathrm{IC}}_{1} / (2\times\uppi )}$$

where P is the effective pressure, T is the wall tension and IC_1_ the internal circumference of the vessel after the normalization procedure. Effective pressure was subsequently converted to mmHg.

### Pressure myography

The superior cerebellar artery was used to determine the passive properties of cerebral arteries. The most proximal segment of the right superior cerebellar artery was mounted onto glass cannulas in a pressure myograph setup. Both ends were secured on the cannulas by nylon sutures. Segments were straightened by adjusting the position of the cannulas at an intraluminal pressure of 100 mmHg. Artery segments were then equilibrated to 37 °C for 15 min in Ca^2+^-free MOPS-buffered PSS containing 1 · 10^–4^ mol/L papaverine (Sigma-Aldrich). A charge-coupled device (CCD) camera connected to an inverted microscope (Nikon) was used to visualise the vessel. Inner and outer diameters were recorded using an in-house written program in MATLAB. The diameter of the superior cerebellar artery was assessed by determining the diameters at an increasing pressure from 1 to 150 mmHg and subsequent decreasing pressure from 150 to 1 mmHg using steps of 25 mmHg of 2 min each. Diameters were subsequently averaged over the two measurements. Distensibility was calculated as (1/ΔP) × (Δd/d) = fractional change in lumen diameter (Δd/d) per change in intraluminal pressure (ΔP). The wall cross-sectional area (CSA) was then calculated at an intraluminal pressure of 1 mmHg as:

$${\text{Wall CSA}}=0.5\uppi ({{\mathrm{d}}_{\mathrm{Outer}}}^{2}- {{\mathrm{d}}_{\mathrm{Inner}}}^{2})$$

where d_Outer_ is the outer diameter and d_Inner_ is the inner diameter of the vessel. Wall-to-lumen ratio was calculated at an intraluminal pressure of 150 mmHg as:

$${\text{Wall-to-lumen ratio } }=\frac{\text{(}{\mathrm{d}}_{\mathrm{Outer}}- {\mathrm{d}}_{\mathrm{Inner}})/2}{{\mathrm{d}}_{\mathrm{Inner}}}$$

### Electron microscopy

A subset of superior cerebellar arteries was used to assess the ultrastructure of these vessels. At the end of the pressure myography experiments, arteries were pressurized to 150 mmHg and fixed in McDowell’s fixative (4% paraformaldehyde, 1% glutaraldehyde in 0.1 M phosphate buffered saline, PBS) for 15 min. After fixation under pressure, vessels were removed from the pressure myograph setup and stored in McDowell’s fixative at 4 °C until use. Arteries were then washed in distilled water for 10 min, postfixed in 1% osmium tetroxide (OsO_4_, Electron Microscopy Sciences) for 1 h, and dehydrated through an ethanol series ranging from 70 to 100%. Samples were immersed in propylene oxide (Merck) for 45 min, and subsequently embedded in Epon 812 epoxy resin (Electron Microscopy Sciences). After polymerization at 60 °C for 2 days, ultrathin cross-sectional sections of 60 nm were cut on a Leica EM UC7 ultramicrotome and were collected on copper formvar-coated slot grids. Sections were stained using uranyl acetate (Merck) and lead citrate (Laurylab).

Superior cerebellar artery sections were examined with a FEI Tecnai G^2^ 12 Spirit BioTWIN transmission electron microscope (FEI Company, Eindhoven, The Netherlands) at 120 kV. Images were acquired using a Veleta side-entry CCD camera (EMSIS, Münster, Germany) at magnifications ranging from × 1200 to × 4800 in order to fully visualise the vessel wall. The total area of the vessel wall, extracellular matrix, and smooth muscle cells were subsequently manually delineated by JdV using FIJI software (version 1.53c) in three different images.

### Immunohistochemistry and neuron quantification

To assess neurodegeneration in the hippocampus and cortex, a slice of the middle part of the brain containing these structures was sectioned coronally (10 µm thick). For fluorescent immunostaining, slides were allowed to equilibrate to room temperature for 30 min, fixed with 3.7% formaldehyde for 20 min, and subsequently rinsed in PBS (pH 7.35). Brain sections were blocked for 1 h at room temperature with 5% normal goat serum, 2% Triton X-100, and 0.2% NaN_3_ (Sigma Aldrich) in PBS. To visualise neuronal nuclei, sections were incubated overnight at 4 °C with the primary antibody mouse anti-NeuN (NeuN, clone A60, dilution 1:200, Cat # MAB377, Millipore) diluted in blocking buffer. Sections were rinsed in PBS and incubated with Cy3-conjugated goat anti-mouse (dilution 1:300, Cat # 115-165-044, Jackson ImmunoResearch Laboratories, Inc.) for 1 h at room temperature. To visualise all nuclei, sections were washed in PBS and incubated in bisbenzimide (dilution 1:100, 3.5 mg/ml, Sigma) for 3 min. After a final rinse, sections were mounted with fluorescent mounting medium (Dako). Detailed images of the cortex and hippocampus were acquired using a confocal laser scanning microscope (Leica TCS SP8 X) with a 20 × objective. The total number of neurons and cells in these images was quantified by intensity thresholding and watershed segmentation to separate adjacent nuclei using FIJI software (version 1.53c).

### Sandwich ELISAs for amyloid-β_1–40_ and amyloid-β_1–42_

Enzyme-linked immunosorbent assay (ELISA) was used to detect and quantify the expression of amyloid-β_1–40_ and amyloid-β_1–42_ in the brain parenchyma. Protein lysates from brain tissue were obtained by dissection of a thick coronal slice, containing the hippocampus. Frozen tissue samples were taken up in cold buffer (50 mM Tris, 5.0 M guanidine, pH 8.0), and after thawing, tissues were mechanically homogenized using a Heidolph Diax900 homogenizer. This was followed by sonication at 10 short pulses with an amplitude of 2.5 (Branson, Danbury, CT, U.S.A.). The homogenate volume was approximately 1 mL buffer per 500 mg brain tissue. To obtain a fully homogenous solution, samples were left at room temperature for 4 h on a shaker. Homogenates were subsequently aliquoted and stored at − 80 °C. Upon assaying, samples were thawed and 5 × diluted in PBS containing a protease inhibitor cocktail (Set I, Cat # 539131, Calbiochem), and centrifuged at 16,000×*g* for 20 min at 4 °C. The supernatant was diluted another 4 times in manufacturer-provided diluent buffer containing the above-mentioned protease inhibitor cocktail. Amyloid-β_1–40_ (Cat # KMB3481, Invitrogen) and amyloid-β_1–42_ (Cat # KMB3441, Invitrogen) assays were subsequently conducted according to the manufacturer’s instructions. In short, 100 µl of samples and standards were added to the appropriate wells of antibody-coated plates, and incubated for 2 h at room temperature. Plates were rinsed four times with wash buffer and 100 µl of detection antibody was subsequently added to each well for 1 h at room temperature. After washing, wells were incubated with 100 µl of HRP-conjugated secondary antibody for 30 min at room temperature. Plates were washed and 100 µl of tetramethylbenzidine (TMB) solution was added to each well. After incubation at room temperature for 30 min, the reaction was stopped by the addition of 100 µl of stop solution to all wells. Absorbance values were subsequently determined at 450 nm using a microplate reader (SpectraMax 250, Molecular devices, Sunnyvale, CA, U.S.A.).

### Neurofilament light

CSF was collected via the cisterna magna at the end of the in vivo imaging procedure and stored at − 80 °C. Upon analysis, samples were thawed and 12.5 × diluted in sample diluent provided with the kit. Neurofilament light (NfL) levels were measured using the Simoa™ NF-light^®^ kit on a Simoa^®^ HD-X analyser (Quanterix Corp., Billerica, MA, U.S.A.), according to the manufacturer’s instructions. During this run, CSF samples were automatically diluted another 4 times, resulting in a final dilution of 50 ×.

### Statistical analysis

Data acquisition and analysis were performed in a blinded manner. All data are depicted as mean ± SEM or median ± interquartile range, where the box contains the values for the 25th and 75th percentile of the data and whiskers extend to the minimum and maximum. Data sets were tested for normality by the use of a QQ plot and Shapiro–Wilk test. Since the neurofilament light measurement showed a clear outlier in the normotensive control group, a Grubbs’ test with a significance level of 0.05 was performed, which resulted in the exclusion of this value. Data for body weight, water intake, blood pressure, endothelial function, and distensibility were analysed using a mixed model effects or repeated measurements two-way ANOVA, followed by Bonferroni’s multiple comparisons test. For non-repeated measurements, differences between groups were determined using one-way ANOVA, followed by Bonferroni’s post hoc test, or by Kruskal–Wallis test with Dunn’s post hoc test. Differences between groups were considered significant when p ≤ 0.05. All statistical analyses were performed using GraphPad Prism Software (version 9.1.0).

## Supplementary Information

Supplementary Figures.

## Data Availability

All data generated or analysed during this study are included in this published article (and its supplementary information files). The datasets generated and/or analysed during the current study are available from the corresponding author on request.
