# Supplementary material for: Effect of long-term antihypertensive treatment on cerebrovascular structure and function in hypertensive rats
Source: Sci Rep. 2023 Mar 1;13:3481. doi: 10.1038/s41598-023-30515-0 (PMC9977931; doi:10.1038/s41598-023-30515-0)
Supplement: Supplementary file 1 — Supplementary Figures. [file 41598_2023_30515_MOESM1_ESM.docx]

**Effect of long-term antihypertensive treatment on cerebrovascular structure and function in hypertensive rats**

Daphne M.P. Naessens^1,2,3^, PhD, Judith de Vos^1,2,3^, Ing, Edo Richard^4,5^, PhD, Micha M.M. Wilhelmus^6,7^, PhD, Cornelis A.M. Jongenelen^6^, Ing, Edwin R. Scholl^8^, Ing, Nicole N. van der Wel^8^, PhD, Johannes A. Heijst^9^, Ing, Charlotte E. Teunissen^7,9,10^, PhD, Gustav J. Strijkers^1^, PhD, Bram F. Coolen^1,2^, PhD, Ed VanBavel^1,2,3^, PhD, Erik N.T.P. Bakker^1,2,3*^, PhD

^1^Amsterdam UMC location University of Amsterdam, Biomedical Engineering and Physics, Amsterdam, the Netherlands. ^2^Amsterdam Cardiovascular Sciences, Microcirculation, Amsterdam, the Netherlands. ^3^Amsterdam Neuroscience, Neurovascular Disorders, Amsterdam, the Netherlands. ^4^Amsterdam UMC location University of Amsterdam, Public and Occupational Health, Amsterdam, the Netherlands. ^5^Department of Neurology, Donders Institute for Brain, Cognition and Behaviour, Radboud University Medical Center, Nijmegen, The Netherlands. ^6^Amsterdam UMC location Vrije Universiteit Amsterdam, Anatomy and Neurosciences, Amsterdam, the Netherlands. ^7^Amsterdam Neuroscience, Neurodegeneration, Amsterdam, the Netherlands. ^8^Amsterdam UMC location University of Amsterdam, Medical Biology, Electron Microscopy Center Amsterdam, Amsterdam, the Netherlands. ^9^Amsterdam UMC location Vrije Universiteit Amsterdam, Neurochemistry Laboratory, Clinical Chemistry, Amsterdam, the Netherlands. ^10^Amsterdam Neuroscience, Neuroinfection & -inflammation, Amsterdam, the Netherlands.

**Supplementary figures**

**
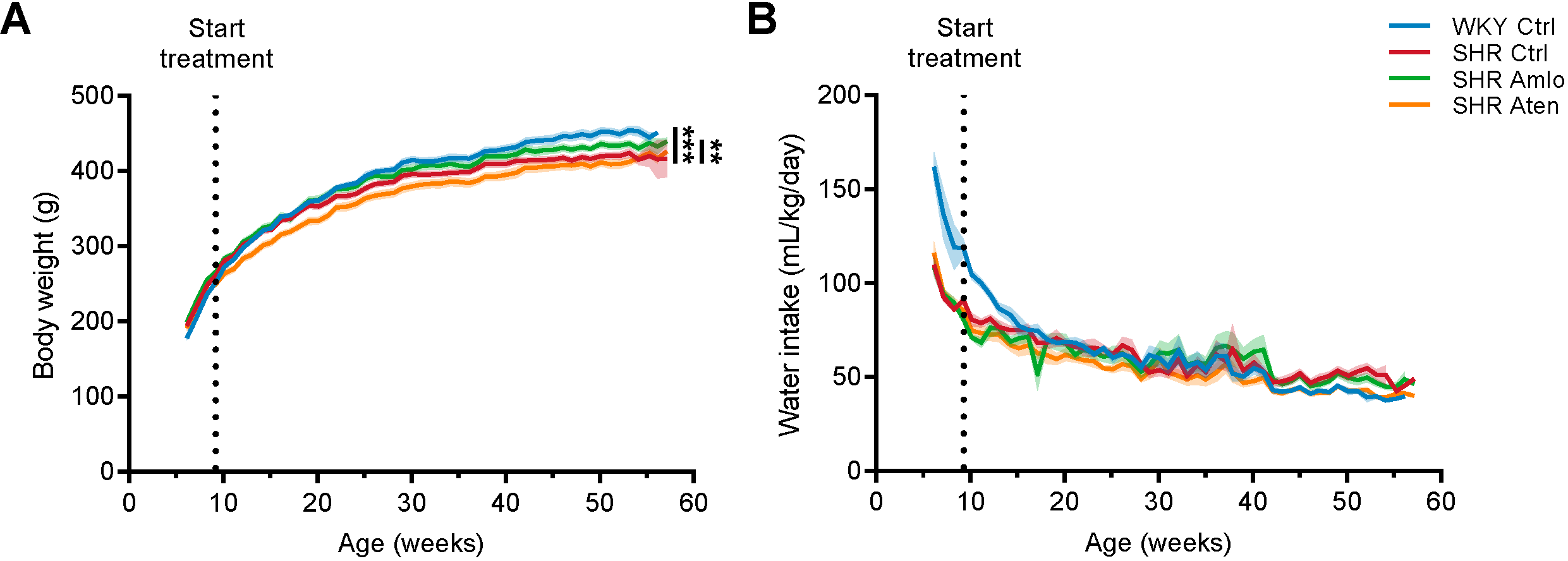
**

**Supplementary figure 1. Body weight and water intake before and during treatment with antihypertensive drugs.** Body weight (**A**) and water intake (**B**) were monitored weekly. Small differences in growth rate were observed between groups during treatment with antihypertensive drugs. Atenolol treated rats appeared to be smaller in comparison to amlodipine treated SHR and WKY. Water intake was higher in WKY at a young age, but gradually decreased to a level similar to SHR. n = 12 for WKY Ctrl and SHR Amlo, and n = 11 for SHR Ctrl and SHR Aten. ***p ≤ 0.001, **p ≤ 0.01 (A and B: mixed-effects model with Bonferroni correction).


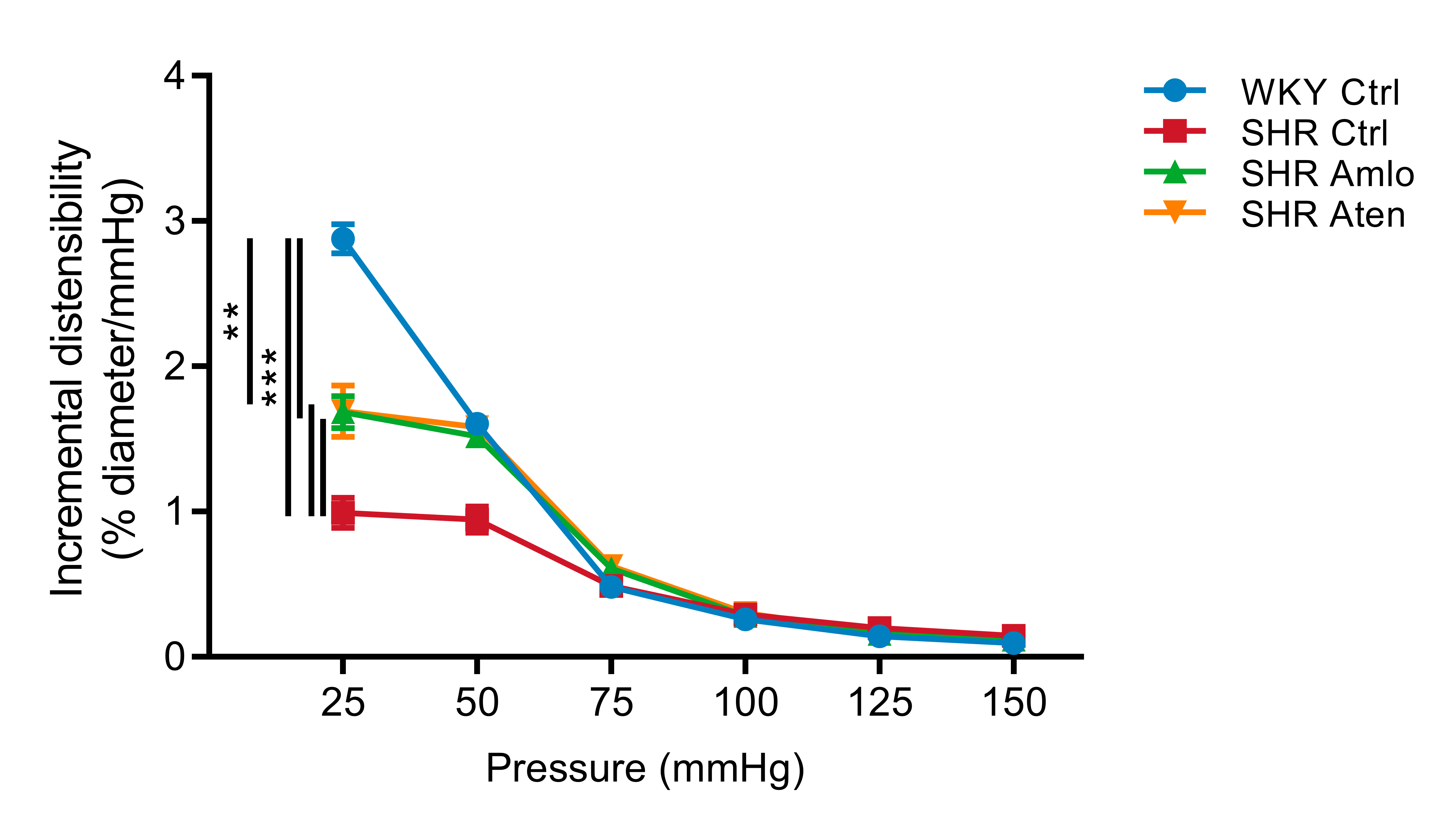


**Supplementary figure 2. Incremental distensibility of the superior cerebellar artery.** Diameter of the superior cerebellar artery was determined by measurement of the intraluminal diameter over a pressure range of 1-150 mmHg, from which the distensibility was calculated. Arteries from untreated SHR showed a smaller distensibility when compared to control WKY. Amlodipine and atenolol treatment increased the distensibility to the same extent, but did not normalize to normotensive levels. n = 12 for WKY Ctrl and SHR Amlo, and n = 11 for SHR Ctrl and SHR Aten. ***p ≤ 0.001 (two-way RM ANOVA with Bonferroni correction).

**
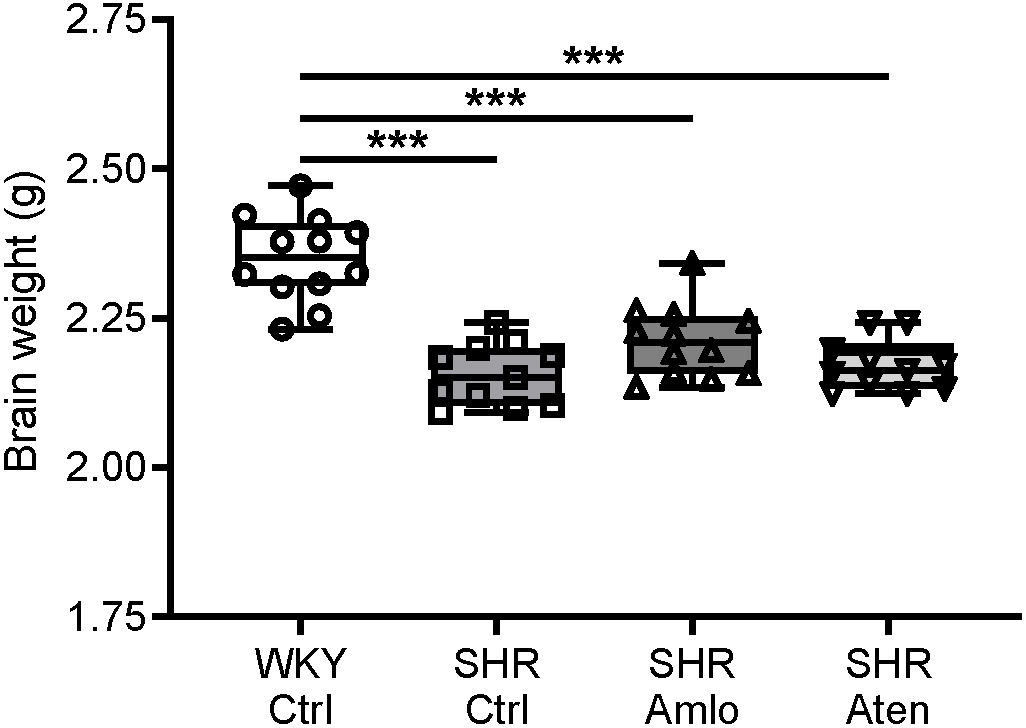
**

**Supplementary figure 3. Brain wet weight.** Brain weights were significantly lower in untreated and treated SHR when compared to normotensive controls. n = 12 for WKY Ctrl and SHR Amlo, and n = 11 for SHR Ctrl and SHR Aten. ***p ≤ 0.001 (one-way ANOVA with Bonferroni correction).
